# Longitudinal association between the dynamic nature of depression with lower urinary tract symptoms suggestive of benign prostatic hyperplasia (LUTS/BPH)

**DOI:** 10.1186/s12889-024-18618-3

**Published:** 2024-04-23

**Authors:** Zonglan Xie, Xuebin Liu, Zhigang Hu, Chuanjiang Dong

**Affiliations:** 1 Department of Urology, The First Dongguan Affiliated Hospital, Guangdong Medical University, Jiaoping Road No. 42, 523710 Dongguan, People’s Republic of China; 2 Department of Evidence Based Medicine Center, The First College of Clinical Medicine Science, China Three Gorges University, 443003 Yichang, China

**Keywords:** LUTS/BPH, Depression, Charls, Middle-aged and older adults

## Abstract

**Background:**

Depression is associated with an increased risk of lower urinary tract symptoms suggestive of benign prostatic hyperplasia (LUTS/BPH). Whether the dynamic nature of depression affects the incidence of LUTS/BPH remains unknown. A four-year cohort study based on the China Health and Retirement Longitudinal Study (CHARLS) was conducted to assess their association.

**Methods:**

This study included 3433 Chinese men from the CHARLS 2011, representative of > 95 million individuals. All eligible individuals underwent three assessments of LUTS/BPH and depression in 2011, 2013 and 2015. The dynamic nature of depression was classified as acute depression with remission, acute depression with recurrence, or chronic major depression. Weighted, generalized additive analyses with three binomial models were used to investigate the relationship between LUTS/BPH and the dynamic nature of depression.

**Results:**

During the four-year follow-up, 11.5% (95% confidence interval [95% CI] = 9.5-13.3%) of Chinese men were diagnosed with newly incident LUTS/BPH. Meanwhile, there were 60.6% (95% CI = 58.5-62.7%) of the individuals without depression and 8.9% (95% CI = 7.9-10%) of the individuals with chronic major depression. A total of 25.1% (95% CI = 23.4-26.9%) and 5.4% (95% CI = 4.6-6.3%) of the individuals were categorized as acute depression with remission and recurrence. After weighted, adjusted all included confounding risk factors, chronic major depression (RR = 1.63, 95% CI = 1.14–2.33, *P* < 0.01) but not acute depression with remission (RR = 1.2, 95% CI = 0.92–1.56, *P* = 0.18) and recurrence (RR = 1.32, 95% CI = 0.82–2.10, *P* = 0.26) significantly increased the incidence of LUTS/BPH compared with no depression. The subgroup analysis showed that the above relationships appeared to be evident among Chinese men < 60 years.

**Conclusions:**

Our results suggest that the dynamic nature of depression has a different effect on the incidence of LUTS/BPH. The monitoring and treatment of depression are important in preventing LUTS/BPH.

**Supplementary Information:**

The online version contains supplementary material available at 10.1186/s12889-024-18618-3.

## Introduction

Benign prostatic hyperplasia (BPH), defined as the unregulated enlargement of the prostate gland, becomes highly prevalent as the population ages [1]. An epidemiological survey involving 8563 Chinese men demonstrated that the prevalence of BPH is 11.9% in individuals aged ≥ 45 years and is 22.7% in those aged ≥ 70 years [2]. Lower urinary tract symptoms (LUTS) secondary to BPH not only affect quality of life, sexual function, and genitourinary health [1], but also increase the risk of falls [3, 4], Alzheimer’s disease [5], all-cause dementia [5] and mortality [3, 4]. Moreover, the use of 5-alpha reductase inhibitors and ɑ-blockers for treating BPH is associated with an increased risk of cardiac failure [6] and contributes directly to polypharmacy. In 2000, the economic burden caused by BPH was estimated to be more than $4 billion per year, which was expected to increase to over $15 billion by 2020 [7]. Therefore, energetic risk screening and prevention methods for LUTS/BPH among elderly men are needed.

Depression is a common and important health problem in the elderly population. Approximately 15–18% of the individuals will experience depression in their lifetime [8], while 43% of women and 30% of men aged over 45 years are regarded as having depression, according to a national survey in China [9]. According to the dynamic nature of depression, researchers have divided depression into three types: acute depression with remission, acute depression with recurrence, or chronic major depression [10]. Approximately 20 to 30% of depressive disorders might be categorized as chronic major depression with depressive symptoms that last for at least 2 years [11]. Compared with acute depression with remission and recurrence, chronic major depression was associated with earlier onset (before 21 years of age) [11], higher comorbidity rates [11], greater somatic morbidity [11], longer delays in treatment [11] and poorer outcomes [12]. Recurrent depression but not acute depression with remission was associated with significantly greater risks of diabetes and cardiovascular disease in a cross-sectional and prospective study [13]. In comparison, chronic major depression among the three patterns might lead to more severe health consequences, followed by acute depression with recurrence and acute depression with remission [14]. Depression and LUTS/BPH share the same risk factors, including high education, poor living conditions, low annual household consumption, reduced sleep duration and multimorbidity [15]. Emerging evidence has shown that depression is an independent risk factor for LUTS/BPH [15–18]. However, the cross-sectional design of the study limited the ability to determine the causal relationship between depression and LUTS/BPH. In addition, no study has explored the association between LUTS/BPH and the dynamic nature of depression. Epidemiological studies on mental health should fully consider the role of depressive patterns in studying the correlation between depression and chronic diseases [19].

In this study, it was hypothesized that patients with chronic major depression would have a greater risk of developing LUTS/BPH due to the more severe effect of chronic major depression on somatic comorbidities and morbidity. We extracted study data from the China Health and Retirement Longitudinal Study (CHARLS) from 2011 to 2015 and evaluated the dynamic nature of depression and the incidence of LUTS/BPH during a four-year follow-up. By using weighted, generalized additive analysis with three binomial models based on cohort data, we clearly explored the relationship between different patterns of depression and incident LUTS/BPH.

## Methods

### Study population

The CHARLS, initially conducted between June 2011 and March 2012, is a nationally representative longitudinal study of Chinese individuals aged ≥ 45 years that involves the examination of social, economic, and health circumstances related to the rapid ageing of the population in China. The multiparty probability- proportional-to-size (PPS) sampling technique was used to choose the study sample, and 150 county-level units were selected from 28 provinces. The first national survey for the CHARLS included 17,708 individuals who provided face-to-face computer-assisted personal interview data and 13,978 individuals who underwent anthropometric and physical measurements. The CHARLS was followed every 2 years and increased by new individuals. The National School of Development of Peking University in China was responsible for maintaining all study data and collected written informed consent from all individuals. The CHARLS was approved by the Biomedical Ethics Review Committee of Peking University. A more detailed description of the CHARLS has been reported elsewhere and in the following link: http://charls.pku.edu.cn/en/ [20].

### Definition of LUTS/BPH

BPH is a term defining the typical histological pattern of the disease. However, not all men with histological BPH seek medical care and require treatment. When BPH is associated with LUTS, the individuals customarily go to the hospital and are diagnosed with LUTS/BPH. According to the CHARLS, the diagnosis of BPH was based on a positively self-reported answer to the following question: “Have you ever been diagnosed with a prostate illness, such as prostate hyperplasia, excluding prostate cancer?”. Individuals with asymptomatic BPH cannot be evaluated. Therefore, “LUTS attributed to BPH” was abbreviated as LUTS/BPH and was used in this study with reference to previous studies [2, 15, 18].

### Diagnosis and dynamic nature of depression

The Center for Epidemiological Studies Depression Scale (CES-D) was used to assess whether the individuals had depression according to the CHARLS. The CES-D consists of ten questions, and every question has four answers, including “rarely”, “some days” (1–2 days), “occasionally” (3–4 days), and “most of the time” (5–7 days). The scores of the four answers ranged from 0 to 3 points; thus, the total score on the CES-D ranged from 0 to 30 points. A previous study also revealed that the CES-D has adequate reliability and validity for assessing depression in middle-aged and elderly Chinese community-dwelling individuals [21]. A total CES-D score ≥ 12 was regarded as indicative of depression according to previous studies [21–24].

All individuals took three self-assessments about depression according to the CHRALS 2011 (baseline), 2013 (follow-up 1), and 2015 (follow-up 2). We divided the changes in depressive symptoms into four groups: baseline yes and follow-up yes (Group 1), baseline no and follow-up yes (Group 2), baseline yes and follow-up no (Group 3), and baseline no and follow-up no (Group 4). Those with no depression from 2011 to 2015 (Group 4) composed the control group. Adults who experienced a depressive episode with a duration less than two years were defined as having acute depression with remission [10]. Therefore, the individuals in Group 3 (baseline yes and follow-up no) were considered to have acute depression with remission. Individuals in Group 2 had three conditions: follow-up 1 yes, follow-up 2 no; follow-up 1 no, follow-up 2 yes; and follow-up 1 yes, follow-up 2 yes. Adults who had depression for more than two years were regarded as having chronic major depression [10]. Therefore, the two previous conditions of Group 2 were classified as acute depression with remission, whereas the third condition of Group 2 was regarded as chronic major depression. Baseline yes, follow-up 1 no and follow-up 2 yes in Group 1 were defined as acute depression with recurrence, while other conditions in Group 1 were considered chronic major depression.

### Covariates

Some potentially confounding variables were used to adjust for the association between incident LUTS/BPH and the dynamic nature of depression. Previous studies [2, 15, 18] have demonstrated that LUTS/BPH and depression share the same risk factors, which include age, education level, night sleep duration and chronic comorbidities. Additionally, body mass index, smoking status, and alcohol consumption were found to be associated with the incidence of LUTS/BPH [2, 18]. Therefore, the variables included in this study included demographic characteristics (age, residential region, marital status, education level, and body mass index), physical/behavioural factors (smoking status, alcohol consumption status, night sleep duration, napping status, and difficulty scores of mobility activities), and health conditions (number of chronic comorbidities, disability status, accidents, and falls).

According to our previous study [25], body mass index was divided into four groups: underweight (< 18.5 kg/m^2^), normal (18.5 to < 24.0 kg/m^2^), overweight (24.0 to < 28.0 kg/m^2^), and obese (≥ 28.0 kg/m^2^). The difficulty score of mobility activities was found to have a mediating effect on the correlations between depression and chronic diseases [26], which was listed as a potentially confounding factor. The scores of mobility activities were defined as a binary variable (yes vs. no), including seven items: walking 100 m, climbing several flights of stairs, getting up from a chair, stooping or kneeling or crouching, extending arms up, lifting 5 kg, and picking up a small coin. Chronic comorbidity in the CHARLS comprised fourteen self-reported physician-diagnosed diseases (hypertension, dyslipidaemia, hyperglycaemia, cancers, chronic lung disease, liver disease, heart disease, stroke, kidney disease, digestive disease, emotional or nervous problems, memory-related disease, arthritis, and asthma). The number of chronic comorbidities was classified into four groups: 0, 1, 2, and ≥ 3. Disability included physical disabilities, brain damage/mental retardation, vision problems, hearing problems, and speech impediments. If individuals had one of the five abovementioned disabilities, they were categorized into the disability group. More detailed groups of all variables were shown in Table 1.

**Table 1:** The characteristics with weighted data of study population in the China health and retirement longitudinal study

	No depression	Acute depression with mission	Acute depression with recurrence	Chronic major depression	*P*
Weight data(%)	57,710,850 (60.6)	23,919,933 (25.1)	5,121,291 (5.3)	8,459,599 (8.9)	
Age(years)	58.5 ± 8.8	58.4 ± 8.4	57.9 ± 7.8	59.6 ± 8.2	0.030
**Age group**					< 0.01
< 60 years	58 (54.5–61.4)	56.5 (52.9–59.9)	57.8 (50-65.1)	48.9 (43.8–54.8)	
≥ 60 years	42 (38.6–45.5)	43.5 (40.1–47.1)	42.2 (34.9–50)	51.1 (45.2–56.2)	
**Urban/Rural**					< 0.001
Urban	56.1 (52.5–59.6)	68.9 (65.3–72.2)	74.7 (67-81.1)	67.6 (61.5–73.1)	
Rural	43.9 (40.4–47.5)	31.1 (27.8–34.7)	25.3 (18.9–33)	32.4 (26.9–38.5)	
**Education levels**					
Under elementary school	52.2 (48.9–55.5)	61.1 (57.6–64.6)	66.7 (62.73.1)	67.8 (62-73.1)	0.008
Elementary and middle school	41 (38–44)	35.4 (32-38.9)	30.3 (23.6–38)	29.5 (24.4–35.1)	
High school or above	6.9 (4.8–9.7)	3.5 (2.4-5.0)	3 (1.1-8.0)	2.8 (1.2–6.3)	
**Married status**					< 0.001
Current unmarried	6.5 (5.3–7.9)	8 (6.4–7.1)	10.4 (6.6–16)	11.3 (8-15.8)	
Current married	93.5 (92.1–94.7)	92 (89.9–93.6)	89.6 (84-93.4)	88.7 (84.2–92)	
**Body mass index category**					0.007
Underweight	4.4 (3.5–5.5)	7.1 (5.4–9.2)	5.6 (3.2–9.6)	10.6 (7.6–14.6)	
Normal	54.9 (51.4–58.3)	61.6 (58.1–65)	66.4 (59-73.1)	57.9 (51.9–63.6)	
Overweight	29.3 (26.4–32.4)	23.5 (20.7–26.6)	21.8 (16.2–28.7)	24.4 (19.6–30)	
Obese	11.4 (8.4–15.2)	7.8 (6.0-10.1)	6.2 (3.6–10.4)	7.1 (4.4–11.2)	
**Smoking**					
Never	27.6 (24.7–30.6)	25.2 (22.2–28.5)	27.5 (21-35.1)	19.7 (15.6–24.6)	< 0.001
Ever	16.8 (13.7–20.5)	13.3 (11-15.9)	14.2 (9.6–20.5)	16.8 (12.6–22)	
Current	55.6 (52.1–59)	61.5 (58-64.9)	58.3 (50.4–65.8)	63.5 (57.6–69)	
**Drinking alcohol**					0.03
More than once a month	49.3 (46-52.6)	41.6 (28.2–45.1)	43.4 (35.9–51.1)	41.1 (35.5–46.9)	
Less than once a month	12.1 (7.1–14.3)	13.3 (10.9–16)	8.4 (4.6–14.8)	10.2 (4.6–14.8)	
Never	38.6 (35.6–41.8)	45.1 (41.6–48.7)	48.2 (40.5–56)	48.8 (42.9–54.6)	
**Difficult mobility**					< 0.001
No	56.3 (53-59.5)	40.2 (36.7–43.7)	16.8 (12.2–22.8)	27.6 (22.6–33.3)	
Yes	43.7 (40.5–47)	59.8 (56.3–63.3)	83.2 (77.2–87.8)	72.4 (66.7–77.4)	
**Night sleep duration**					< 0.001
< 6 h	17.8 (15.8–19.9)	31.3 (28.1–34.7)	40.6 (33.2–48.4)	39.7 (24.1–45.5)	
6–6.99 h	22.9 (20.2–25.8)	21.4 (18.6–24.4)	19.9 (14.2–27)	21.7 (17.1–27)	
7–8.9 h	49.9 (46.6–53.3)	38.9 (35.5–42.5)	36 (28.8–43.8)	33.5 (28.1–39.3)	
≥ 9 h	9.5 (6.6–13.3)	8.4 (6.6–10.6)	3.6 (1.8–6.9)	5.2 (3.2–8.4)	
**Napping**					0.06
0 h	41.7 (38.5–44.9)	44.7 (41.1–48.3)	44.9 (37.4–52.7)	43.8 (38.2–49.7)	
0.1–1 h	19.2 (16.6–22.2)	18.5 (15.9–21.4)	13.4 (9-19.4)	13.2 (9.8–17.4)	
> 1 h	39.1 (35.8–42.4)	36.8 (33.5–40.3)	41.7 (34.2–49.6)	43 (37.2–48.9)	
**The number of chronic multimorbidity**					< 0.001
0	40.8 (37.9–43.9)	36.2 (32.9–39.7)	26.5 (20.3–33.7)	22.4 (17.9–27.7)	
1	29.9 (27.1–33)	29.8 (36.6–33.2)	30.8 (24.1–38.4)	30.5 (25.4–36.1)	
2	18.3 (15.2–21.9)	19.2 (16.7–22.1)	23.3 (17–31)	20 (15.8–25.1)	
≥ 3	10.9 (8.6–13.8)	14.8 (12.5–17.4)	19.5 (14.1–26.3)	27.1 (22.1–32.7)	
**Disabilities**					< 0.001
No	87.7 (86-89.2)	83.5 (80.7–85.9)	69.6 (62-76.4)	72.2 (66.7–77.2)	
Yes	12.3 (10.8–14)	16.5 (14.1–19.3)	30.4 (23.6–38)	27.8 (22.8–33.3)	
**Accident**					0.086
No	87 (84.2–89.4)	87.7 (85.1–90)	85.4 (78.6–90.3)	82.2 (77.3–86.3)	
Yes	13 (10.6–15.8)	12.3 (10-14.9)	14.6 (9.7–21.4)	17.8 (16.7–22.7)	
**Fallen down**					< 0.001
No	89.2 (85.5–92.1)	88.1 (85.6–90.2)	79.7 (72.5–85.4)	80.5 (75.5–84.7)	
Yes	10.8 (7.9–14.5)	11.9 (9.8–14.4)	20.3 (14.6–27.5)	19.5 (15.3–24.5)	
**LUTS/BPH**					0.009
No	89 (86.2–91.3)	89.1 (86.8–91.1)	85.6 (78.3–90.7)	84.8 (80.2–88.6)	
Yes	11 (8.7–13.8)	10.9 (8.9–13.2)	14.4 (9.3–21.7)	15.2 (11.4–19.8)	
**Depressive symptom scores (CED-S10)**					< 0.001
Year 2011	6.8 ± 2.5	10.7 ± 4.3	15.0 ± 2.9	13.6 ± 4.7	< 0.001
Year 2013	5.3 ± 2.9	7.7 ± 4.4	7.2 ± 2.9	15.4 ± 3.2	< 0.001
Year 2015	5.8 ± 2.7	9.2 ± 4.6	15.2 ± 3.3	13.7 ± 5.8	0.06

*Note* LUTS/BPH, lower urinary tract symptoms suggestive of benign prostatic hyperplasia

Weighted means and proportions were displayed

### Inclusion and exclusion criteria

All eligible individuals were men and had diagnostic data about LUTS/BPH at CHRALS 2011, 2013, and 2015. Finally, individuals who required three assessments of the CES-D score during three follow-ups were included. Individuals with LUTS/BPH in the CHARLS 2011 were excluded from the study. In addition, individuals who had no data for the abovementioned confounding factors in the CHARLS 2011 were also excluded.

### Statistical analyses

First, the study population was categorized into four groups according to the dynamic nature of depression. The unweighted frequencies, means and proportions of the study population are shown in Table S1. Categorical variables are presented as counts and percentages (%), and differences in categorical variables among the four groups were compared by using the chi-squared test. This study compared continuous variables through means and standard deviations (SDs) and compared two groups by using one-way analysis of variance (ANOVA) for normally distributed continuous variables and the Mann–Whitney U test for skewed continuous variables. We applied the chi-square goodness of fit test and the Kolmogorov‒Smirnov test to explore the normality of the distribution of variables. The weighted means and proportions with 95% confidence intervals (CIs) of all included variables are shown in Table 1. Second, we applied weighted, generalized additive analysis with three binomial models to determine the associations between LUTS/BPH and the dynamic nature of depression. Compared with repeated measures analysis of variance and traditional linear mixed models, generalized additive analysis relaxes the linearity assumption, allows the data to be fit by the model, and permits incomplete observations and different correlation structures. Accordingly, generalized additive analysis is considered an excellent choice for analysing longitudinal data with nonlinear trends [27]. Model 1 was adjusted for demographic characteristics of the included individuals (including age, residential region, marital status, education level, and body mass index), Model 2 was adjusted for demographic characteristics and behavioural factors (including smoking status, alcohol consumption, night sleep duration, napping status, and difficulty score for mobility), and Model 3 included health conditions (including the number of chronic comorbidities, disability, accidents, and falls) on the basis of Model 2. The tolerance and variance inflation factor (VIF) was used to examine the problem of collinearity of all variables. When the tolerance of a variable was less than 0.1 with a VIF ≥ 5, it was excluded from the adjusted model. Finally, one predefined subgroup analysis stratified by age (≥ 60 or < 60 years) was performed to determine whether age could affect the relationship between the dynamic nature and risk of LUTS/BPH according to a previous study [28]. We completed all the statistical analyses via Empower (R) software (www.empowerstats.com; X&Y Solutions, Inc., Boston, MA, USA) [24, 26]. Relative risks (RRs) with 95% CIs were used to represent the strength of all analyses, and a two-tailed *p* < 0.05 was considered to indicate statistical significance.

## Results

### Characteristics of the study cohort

**Fig. 1:**
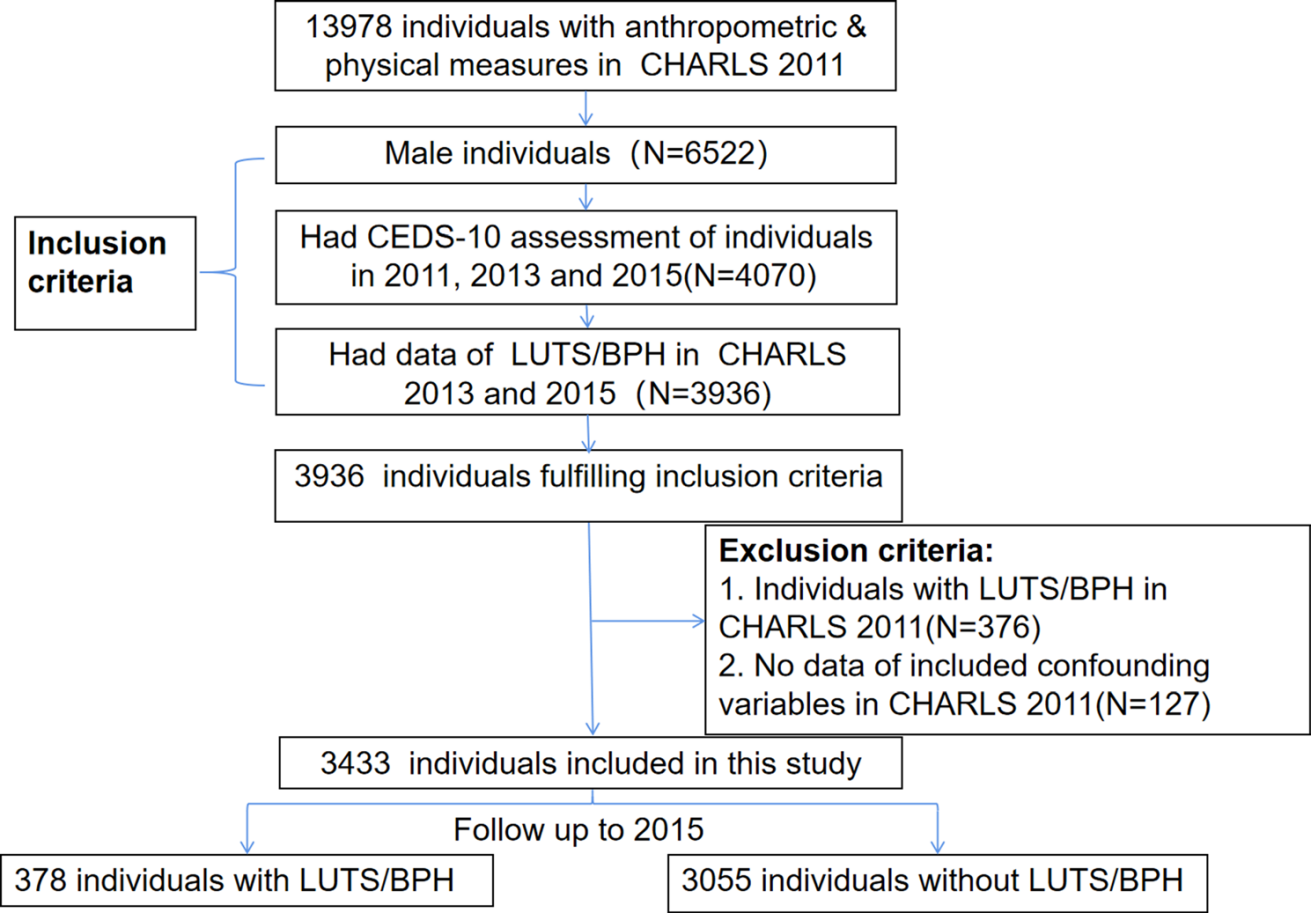
Flow diagram of study population

A total of 3433 unique Chinese middle-aged and elderly men without LUTS/BPH were included in the present study (Fig. 1). Considering that sampling weights were incorporated into these analyses, this study population represented more than 95 million individuals. The weighted mean age of the study individuals was 58.6 years (95% CI = 58.1–59 years, SD = 0.2 years). Of this study population, 43.2% (95% CI= 40.9-45.6%) were aged more than 60 years, and 38.7% (95% CI = 36.2-41.3%) lived in urban areas. The proportions of current smokers and never drinkers were 57.9% (95% CI = 55.5-60.3%) and 41.7% (95% CI = 39.5-43.9%), respectively. Approximately 9.8% (95% CI = 7.8-12.2%) of the study population was obese. Approximately 46.6% (95% CI = 44.3-48.8%) and 57.2% (95% CI = 54.9-59.4%) of the cohort experienced a sleep duration less than 7 h and daytime napping, respectively. A total of 52.4% (95% CI = 50.1-54.8%) of the study population had difficulty performing mobility activities. Individuals with ≥ 3 chronic comorbidities accounted for approximately 13.8% (95% CI = 12.1-15.6%) of the study population, and 15.7% (95% CI = 14.4-17.1%) had at least one disability. The prevalence of depression in 2011, 2013, and 2015 was 23.6% (95% CI = 22-25.3%), 14.1% (95% CI = 12.8-15.4%), and 19.4% (95% CI = 17.8 -21%), respectively.

### Prevalence of different dynamic natures of depression

**Fig. 2:**
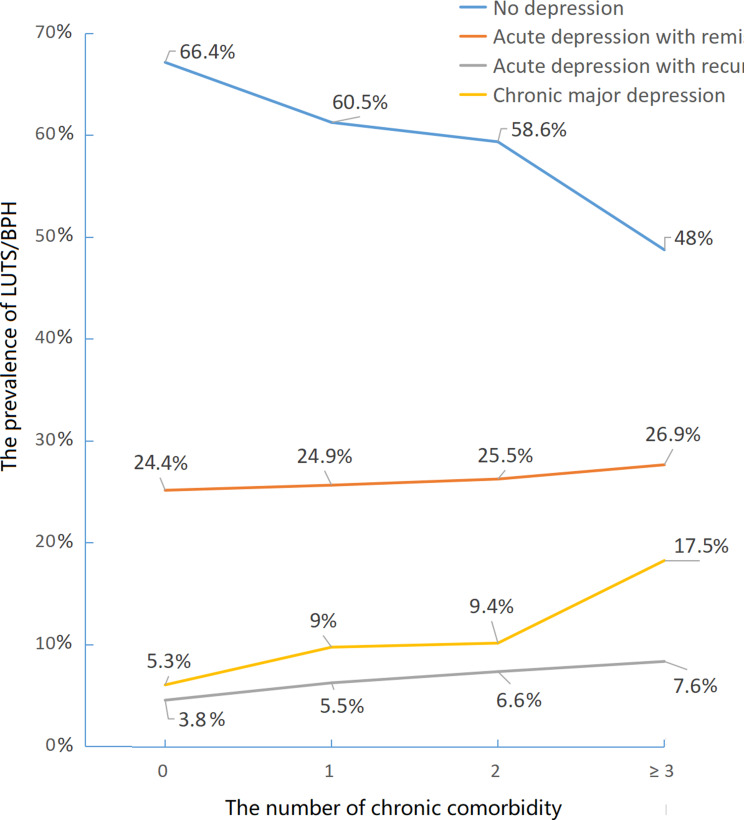
The weighted prevalence of four patterns of depression in different numbers of chronic comorbidity

The weighted and unweighted detailed characteristics of the study population stratified by the dynamic nature of depression are shown in Table 1 and Table S1, respectively. Among Chinese middle-aged and elderly men, 60.6% (95% CI = 58.5 to 62.7%) of individuals had no depression during the four-year follow-up. The weighted overall prevalences of acute depression with remission and recurrence were 25.1% (95% CI = 23.4-26.9%) and 5.4% (95% CI = 4.6- 6.3%), respectively. Approximately 8.9% (95% CI = 7.9-10%) of individuals in this cohort were considered to have chronic major depression. Individuals with chronic major depression had a lower body mass index (weighted average ± SD = 22.6 ± 0.1 kg/m^2^ vs. 23.6 ± 0.1 kg/m^2^) and night sleep duration (weighted average ± SD = 5.9 ± 0.1 h vs. 6.8 ± 0.1 h) and more difficult mobility activities, disability and comorbidity (see Table 1) than those with no depression. Individuals aged ≥ 60 years had a greater weighted prevalence of chronic major depression (10.5% vs. 7.5%, *P* = 0.03) than those aged < 60 years. Individuals who lived in rural areas were more likely to have no depression (68.8% vs. 55.4%, *P* < 0.01) than were those who lived in urban areas. The weighted prevalence of the three dynamic aspects of depression demonstrated a dose-dependent relationship with the number of chronic comorbidities (Fig. 2).

### Incidence of LUTS/BPH

During the four-year follow-up, 378 individuals were diagnosed with LUTS/BPH, with an incidence of 27.4/1000 person-years. The four-year overall weighted incidence of LUTS/BPH was 11.5% (95% CI = 9.9-13.3%). The weighted incidence of LUTS/BPH was 8.7% (95% CI = 8.7-10.3%) in the 45- to 60-year-old group and increased by 15% (95% CI = 12-18.9%) among those aged 60 years and older. The incidence of LUTS/BPH gradually increased with the increasing number of chronic comorbidities.

### Associations between LUTS/BPH and the dynamic nature of depression

**Fig. 3:**
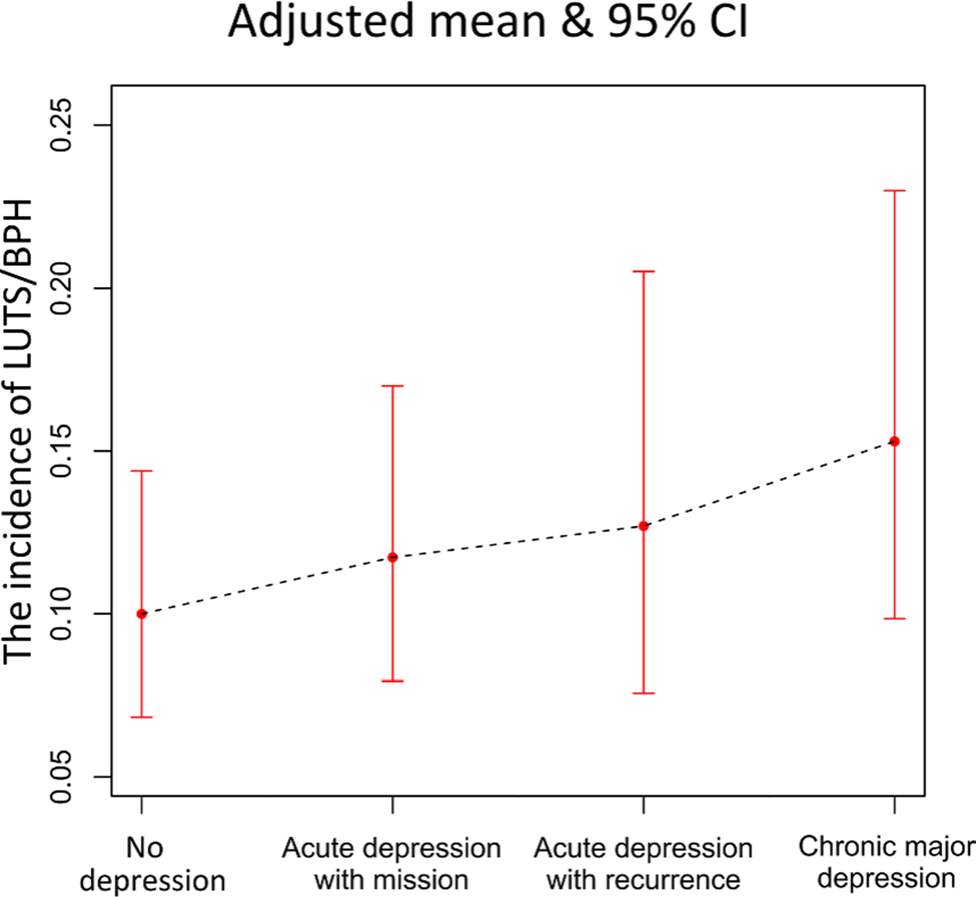
The adjusted incidence of lower urinary tract symptoms suggestive of benign prostatic hyperplasia (LUTS/BPH) in different dynamic nature of depression

The weighted incidences of LUTS/BPH in individuals with acute depression with recurrence and those with chronic major depression were 14.4% (95% CI = 9.3 -21.7%) and 15.2% (95% CI = 11.4-19.8%), respectively, which were greater than those in individuals with no depression (11%, 95% CI = 8.7-13.8%) (*P* < 0.01). All three models demonstrated that compared with no depression, only chronic major depression (RR = 1.63, 95% CI = 1.14–2.33, *P* < 0.01 in Model 3) significantly increased the incidence of LUTS/BPH (Table 2; Fig. 3). After adjusting for all included confounding risk factors, acute depression with remission (RR = 1.2, 95% CI = 0.92–1.56, *P* = 0.18) and recurrence (RR = 1.32, 95% CI = 0.82–2.10, *P* = 0.26) had increasing but not significantly different trends in the development of LUTS/BPH than no depression (see Table 2; Fig. 3).

**Table 2:** The associations between the dynamic nature of depression with incident lower urinary tract symptoms suggestive of benign prostatic hyperplasia on weighted data

	Model 1	*P*	Model 2	*P*	Model 3	*P*
No depression	Ref		Ref		Ref	
Acute depression with mission	1.24 (0.96–1.60)	0.1	1.22 (0.94–1.58)	0.14	1.18 (0.91–1.54)	0.21
Acute depression with recurrence	1.41 (0.89–2.23)	0.14	1.36 (0.85–2.18)	0.2	1.30 (0.81–2.09)	0.28
Chronic major depression	1.77 (1.26–2.50)	< 0.01	1.74 (1.22–2.47)	< 0.01	1.59 (1.11–2.28)	0.01

Model 1 adjusted for the following variables: age, residential regions, married status, education levels, and body mass index

Model 2 adjusted for the following variables: age, residential regions, married status, education levels, body mass index, smoking, drinking alcohol, night sleep duration, napping, and difficulty scores of mobility activities

Model 3 adjusted for the following variables: age, residential regions, married status, education levels, body mass index, smoking, drinking alcohol, night sleep duration, napping, difficulty scores of mobility activities, the number of chronic comorbidity, disability, accident, and fallen down

**Fig. 4:**
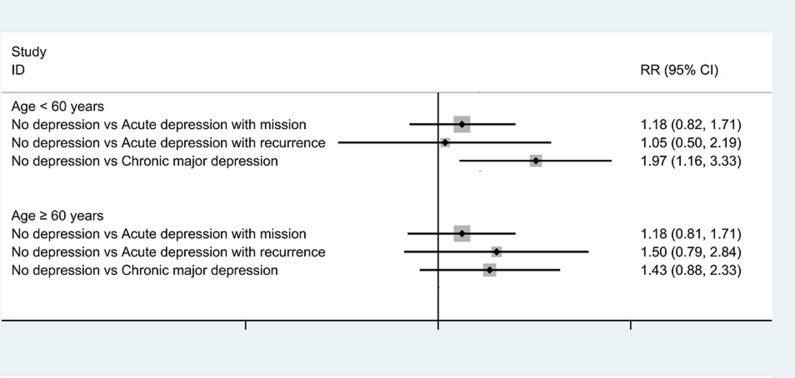
The associations between different dynamic nature of depression with the adjusted incidence of lower urinary tract symptoms suggestive of benign prostatic hyperplasia (LUTS/BPH) stratified by age in model 3

To further assess the association between LUTS/BPH and the dynamic nature of depression, we conducted one subgroup analysis stratified by age (≥ 60 or < 60 years). The effect of chronic major depression on the incidence of LUTS/BPH appeared to be evident among Chinese men < 60 years (see Fig. 4). However, the interaction test showed that the above relationship was not significantly modified by age (*P* for interaction > 0.10 for all outcomes).

## Discussion

This investigation provided important epidemiological data about the prevalence of different patterns of depression and the incidence of LUTS/BPH based on a nationally representative cohort study. In this four-year longitudinal study of Chinese middle-aged and elderly men from 28 provinces, the most crucial finding was that the different dynamic natures of depression had different impacts on the development of LUTS/BPH. Among the three dynamic forms of depression, only chronic major depression significantly increased the incidence of LUTS/BPH compared with no depression, which was more evident among men aged < 60 years but not among those aged ≥ 60 years. These findings are representative of > 90 million middle-aged and elderly adults without LUTS/BPH. This study population is one of the largest samples of middle-aged and elderly individuals in terms of LUTS/BPH and the dynamic nature of depression to date.

Since 1980, many regional studies have reported epidemiological data on depressive disorders among the general population in China [29]. Some nationally representative studies based on the CHARLS demonstrated that the prevalence of depression among middle-aged and elderly adults was approximately 20–30% [2, 15, 18, 28]. However, these studies did not consider the dynamic nature of depression. Our study not only reported the weighted prevalence of depression in 2011, 2013 and 2015 but also reported the weighted prevalence of three dynamic aspects of depression. During the four-year follow-up, 8.9% of Chinese men were diagnosed with chronic major depression, while 25.1% and 5.4% of Chinese men were diagnosed with acute depression with remission and recurrence, respectively. Approximately one in five individuals with depression might develop chronic major depression, which was similar to the findings of previous studies [11–13]. Ageing, short night sleep duration, low body mass index, disability, and high difficulty performing mobility activities were more strongly associated with chronic major depression. Notably, the number of chronic comorbidities displayed a dose-dependent relationship with the weighted prevalence of all three patterns of depression. When the number of chronic comorbidities was three or more, the weighted prevalences of chronic major depression and acute depression with recurrence were three times and two times greater, respectively, than when there was no comorbidity (see Fig. 3). The major consequences of multimorbidity might include disability, functional decline and poor quality of life [30], increasing the risk of depression [15].

The incidence of LUTS/BPH ranges from 9 to 41 per 1000 person-years in some countries in the West and North America [31], while a study from Korea reported a 21 per 1000 person-years incidence of BPH [32]. Studies from China reported that the prevalence of LUTS/BPH fluctuates between 8.3% and 13.1% [2, 15, 29]. Data on the incidence of LUTS/BPH are relatively scarce in China. Here, we performed this four-year follow-up cohort study and reported 27.4 per 1000 person-years and 28.7 per 1000 person-years for the unweighted and weighted incidence of LUTS/BPH, respectively. According to previous studies [31, 32], ageing was regarded as an independent risk factor for LUTS/BPH. Compared with a previous study [15], this cohort study also further explored the causal association between chronic comorbidities and the risk of developing LUTS/BPH.

Cross-sectional studies have demonstrated that depression is associated with an increased risk of LUTS/BPH [2, 15] and more severe symptoms of LUTS/BPH [16]. A two-year cohort study based on propensity score matching further confirmed that depression can increase the 2.10-fold (95% CI: 1.48–2.98, *P* < 0.01) risk of developing LUTS/BPH compared with no depression among middle-aged and elderly males [18]. By comparison, our four-year follow-up time was longer, and weighted analyses could represent more than 95 million individuals. More importantly, our study aimed to determine the association between different dynamic factors and new-onset LUTS/BPH. As mentioned above, chronic major depression but not acute depression with remission and recurrence significantly increased the risk of developing LUTS/BPH. Our findings further support the theory that different dynamic natures of depression have different effects on clinical health outcomes. Although the underlying mechanism linking depression and LUTS/BPH has not been fully elucidated, systemic inflammation secondary to depression has been deemed to play an important role in the pathogenesis of LUTS/BPH [18]. Systemic inflammation was found to result in the proliferation of epithelial and stromal prostatic cells followed by LUTS/BPH [33, 34]. In addition, we speculated that chronic comorbidities may mediate the effect of depression on the incidence of LUTS/BPH. Our results suggested that the number of chronic comorbidities showed dose-dependent relationships with both the incidence of chronic major depression and the incidence of LUTS/BPH. Multimorbidity was defined as two or more kinds of chronic comorbidities in an individual [31]. The weighted prevalence of multimorbidity in the chronic major depression group was greater (47.1% vs. 29.2%, *P* < 0.01) than that in the no depression group. Further studies are required to perform a mediation analysis of depression, multimorbidity, and LUTS/BPH.

Interestingly, our subgroup analysis demonstrated that the effect of chronic major depression on the risk of developing LUTS/BPH appeared to be more evident among Chinese men aged < 60 years, while a relatively lower RR without statistical significance was shown among those aged ≥ 60 years. In comparison, men aged ≥ 60 years were associated with a greater incidence of multimorbidity and a greater incidence of chronic inflammation [28]. Moreover, ageing itself was an independent risk factor for LUTS/BPH. These consequences might partially offset the detrimental effects of chronic major depression on the risk of LUTS/BPH. Further large-sample investigations are warranted to verify our results and explore the cause of this phenomenon.

The main strength of this study was that a nationally representative cohort was used to determine the causal association between the dynamic nature of depression and the incidence of LUTS/BPH based on four-year follow-up data. Certainly, some limitations still occurred in this study. First, we referred to previous studies for the definition of LUTS/BPH [2, 15, 18] and self-reported results rather than objective tests such as prostatic ultrasonography, which may have introduced bias in the diagnosis. Second, some types of selection bias, such as potential volunteer bias and nonresponse bias, should also be considered when interpreting and extrapolating our results. Finally, although we reported the weighted prevalence of three patterns of depression by using a nationally representative study, the results focused on Chinese middle-aged and elderly men but not the general population. Compared with men, women have twice as high a risk of depression [35]. Therefore, large-sample representative surveys are needed to determine the epidemiological data and risk factors for different patterns of depression.

## Conclusions

The findings suggest that different dynamic natures of depression have different effects on the incidence of LUTS/BPH. Early management and intervention to decrease the transition from acute depression to chronic major depression can reduce the risk of developing LUTS/BPH. Ageing and multimorbidity potentially influence the effect of chronic major depression on the incidence of LUTS/BPH, which needs to be validated via further large-sample cohort studies.

### Electronic supplementary material

Below is the link to the electronic supplementary material.

Supplementary Material 1

## Data Availability

The data involving this study were obtained from open CHALRS database. All relevant data are within the paper and its Supporting Information files.
