# Supplementary material for: Longitudinal association between the dynamic nature of depression with lower urinary tract symptoms suggestive of benign prostatic hyperplasia (LUTS/BPH)
Source: BMC Public Health. 2024 Apr 23;24:1121. doi: 10.1186/s12889-024-18618-3 (PMC11040863; doi:10.1186/s12889-024-18618-3)
Supplement: Supplementary file 1 — Supplementary Material 1 [file 12889_2024_18618_MOESM1_ESM.docx]

| Table S1: The characteristics of unweighted study population in the China health and retirement longitudinal study | | | | | |
| --- | --- | --- | --- | --- | --- |
|  | No | Acute depression | Acute depression | Chronic major | P |
|  | depression | with mission | with recurrence | depression |  |
| N | 2021 | 896 | 194 | 322 |  |
| Age(years) | 58.5 ± 8.6 | 58.5 ± 8.4 | 58.4 ± 7.5 | 59.6 ± 8.2 | 0.183 |
| Age group |  |  |  |  | 0.022 |
| < 60 years | 1182 (58.5%) | 506 (56.5%) | 109 (56.2%) | 159 (49.4%) |  |
| ≥ 60 years | 839 (41.5%) | 390 (43.5%) | 85 (43.8%) | 163 (50.6%) |  |
| Urban/Rural |  |  |  |  | <0.001 |
| Urban | 1282 (63.4%) | 636 (71.0%) | 147 (75.8%) | 232 (72.0%) |  |
| Rural | 739 (36.6%) | 260 (29.0%) | 47 (24.2%) | 90 (28.0%) |  |
| Education levels |  |  |  |  | <0.001 |
| Under elementary school | 1049 (51.9%) | 544 (60.7%) | 129 (66.5%) | 222 (68.9%) |  |
| Elementary and middle school | 853 (42.2%) | 320 (35.7%) | 60 (30.9%) | 93 (28.9%) |  |
| High school or above | 119 (5.9%) | 32 (3.6%) | 5 (2.6%) | 7 (2.2%) |  |
| Married status |  |  |  |  | 0.003 |
| Current unmarried | 123 (6.1%) | 74 (8.3%) | 21 (10.8%) | 34 (10.6%) |  |
| Current married | 1898 (93.9%) | 822 (91.7%) | 173 (89.2%) | 288 (89.4%) |  |
| Body mass index category | |  |  |  | <0.001 |
| Underweight | 93 (4.6%) | 62 (6.9%) | 13 (6.7%) | 35 (10.9%) |  |
| Normal | 1170 (57.9%) | 550 (61.4%) | 122 (62.9%) | 193 (59.9%) |  |
| Overweight | 571 (28.3%) | 217 (24.2%) | 44 (22.7%) | 73 (22.7%) |  |
| Obesity | 187 (9.3%) | 67 (7.5%) | 15 (7.7%) | 21 (6.5%) |  |
| Smoking |  |  |  |  | 0.098 |
| Never | 547 (27.1%) | 218 (24.3%) | 51 (26.3%) | 65 (20.2%) |  |
| Ever | 303 (15.0%) | 119 (13.3%) | 28 (14.4%) | 50 (15.5%) |  |
| Current | 1171 (57.9%) | 559 (62.4%) | 115 (59.3%) | 207 (64.3%) |  |
| Drinking alcohol |  |  |  |  | <0.001 |
| More than once a month | 1019 (50.4%) | 384 (42.9%) | 87 (44.8%) | 136 (42.2%) |  |
| Less than once a month | 214 (10.6%) | 113 (12.6%) | 16 (8.2%) | 34 (10.6%) |  |
| Never | 788 (39.0%) | 399 (44.5%) | 91 (46.9%) | 152 (47.2%) |  |
| Difficult mobility |  |  |  |  | <0.001 |
| No | 1117 (55.3%) | 359 (40.1%) | 37 (19.1%) | 86 (26.7%) |  |
| Yes | 904 (44.7%) | 537 (59.9%) | 157 (80.9%) | 236 (73.3%) |  |
| Night sleep duration | |  |  |  | <0.001 |
| < 6h | 363 (18.0%) | 275 (30.7%) | 77 (39.7%) | 131 (40.7%) |  |
| 6-6.99h | 466 (23.1%) | 190 (21.2%) | 38 (19.6%) | 67 (20.8%) |  |
| 7-8.9h | 1017 (50.3%) | 355 (39.6%) | 70 (36.1%) | 106 (32.9%) |  |
| ≥ 9h | 175 (8.7%) | 76 (8.5%) | 9 (4.6%) | 18 (5.6%) |  |
| Napping |  |  |  |  | 0.214 |
| 0h | 826 (40.9%) | 381 (42.5%) | 90 (46.4%) | 146 (45.3%) |  |
| 0.1-1h | 354 (17.5%) | 165 (18.4%) | 25 (12.9%) | 45 (14.0%) |  |
| > 1h | 841 (41.6%) | 350 (39.1%) | 79 (40.7%) | 131 (40.7%) |  |
| The number of chronic multimorbidity | |  |  |  | <0.001 |
| 0 | 861 (42.6%) | 320 (35.7%) | 54 (27.8%) | 73 (22.7%) |  |
| 1 | 606 (30.0%) | 263 (29.4%) | 61 (31.4%) | 99 (30.7%) |  |
| 2 | 356 (17.6%) | 179 (20.0%) | 42 (21.6%) | 64 (19.9%) |  |
| ≥3 | 198 (9.8%) | 134 (15.0%) | 37 (19.1%) | 86 (26.7%) |  |
| Disabilities |  |  |  |  | <0.001 |
| No | 1753 (86.7%) | 740 (82.6%) | 137 (70.6%) | 230 (71.4%) |  |
| Yes | 268 (13.3%) | 156 (17.4%) | 57 (29.4%) | 92 (28.6%) |  |
| Accident |  |  |  |  | 0.005 |
| No | 1784 (88.3%) | 787 (87.8%) | 166 (85.6%) | 262 (81.4%) |  |
| Yes | 237 (11.7%) | 109 (12.2%) | 28 (14.4%) | 60 (18.6%) |  |
| Fallen down |  |  |  |  | <0.001 |
| No | 1807 (89.4%) | 786 (87.7%) | 154 (79.4%) | 258 (80.1%) |  |
| Yes | 214 (10.6%) | 110 (12.3%) | 40 (20.6%) | 64 (19.9%) |  |
| LUTS/BPH |  |  |  |  | 0.025 |
| No | 1819 (90.0%) | 794 (88.6%) | 170 (87.6%) | 272 (84.5%) |  |
| Yes | 202 (10.0%) | 102 (11.4%) | 24 (12.4%) | 50 (15.5%) |  |
| Depressive symptom scores(CED-S10) | | |  |  |  |
| Year 2011 | 6.7 ± 2.5 | 10.8 ± 4.3 | 14.9 ± 2.8 | 13.8 ± 4.6 | <0.001 |
| Year 2013 | 5.4 ± 2.9 | 7.7 ± 4.4 | 7.2 ± 2.8 | 15.4 ± 3.2 | <0.001 |
| Year 2015 | 5.8 ± 2.7 | 9.2 ± 4.6 | 15.3 ± 3.4 | 13.9 ± 5.7 | <0.001 |
| Note: LUTS/BPH, lower urinary tract symptoms suggestive of benign prostatic hyperplasia. | | | | | |
